# Seed-Specific Expression of *AtLEC1* Increased Oil Content and Altered Fatty Acid Composition in Seeds of Peanut (*Arachis hypogaea* L.)

**DOI:** 10.3389/fpls.2018.00260

**Published:** 2018-03-06

**Authors:** Guiying Tang, Pingli Xu, Wenhua Ma, Fang Wang, Zhanji Liu, Shubo Wan, Lei Shan

**Affiliations:** ^1^Shandong Provincial Key Laboratory of Crop Genetic Improvement, Ecology and Physiology, Bio-Tech Research Center, Shandong Academy of Agricultural Sciences, Jinan, China; ^2^College of Life Sciences, Shandong University, Jinan, China; ^3^College of Life Sciences, Shandong Normal University, Jinan, China; ^4^Shandong Cotton Research Center, Shandong Academy of Agricultural Sciences, Jinan, China

**Keywords:** transgenic peanut (*Arachis hypogaea* L.), *Arabidopsis LEC1* (*AtLEC1*) overexpression, seed-specific promoter, fatty acid synthesis, oil accumulation

## Abstract

Peanut (*Arachis hypogaea* L.) is one of the major oil crops and is the fifth largest source of plant oils in the world. Numerous genes participate in regulating the biosynthesis and accumulation of the storage lipids in seeds or other reservoir organs, among which several transcription factors, such as *LEAFY COTYLEDON1* (*AtLEC1*), *LEC2*, and *WRINKLED1* (*WRI1*), involved in embryo development also control the lipid reservoir in seeds. In this study, the *AtLEC1* gene was transferred into the peanut genome and expressed in a seed-specific manner driven by the NapinA full-length promoter or its truncated 230-bp promoter. Four homozygous transgenic lines, two lines with the longer promoter and the other two with the truncated one, were selected for further analysis. The *AtLEC1* mRNA level and the corresponding protein accumulation in different transgenic overexpression lines were altered, and the transgenic plants grew and developed normally without any detrimental effects on major agronomic traits. In the developing seeds of transgenic peanuts, the mRNA levels of a series of genes were upregulated. These genes are associated with fatty acid (FA) biosynthesis and lipid accumulation. The former set of genes included the homomeric ACCase A (*AhACC II*), the BC subunit of heteromeric ACCase (*AhBC4*), ketoacyl-ACP synthetase (*AhKAS II*), and stearoyl-ACP desaturase (*AhSAD*), while the latter ones were the diacylglycerol acyltransferases and oleosins (*AhDGAT1, AhDGAT2, AhOle1, AhOle2*, and *AhOle3*). The oil content and seed weight increased by 4.42–15.89% and 11.1–22.2%, respectively, and the levels of major FA components including stearic acid, oleic acid, and linoleic acid changed significantly in all different lines.

## Introduction

Peanut (*Arachis hypogaea* L.) is the fifth largest source of plant oil in the world, after soybean, rapeseed, cotton, and sunflower (de Paula et al., 2017). Its global production during 2015–2016 and 2016–2017 was 40.42 and 42.77 million tons, respectively (USDA, 2018). The oil contents of its seeds in the current commercial cultivars account for about 50% of seed fried weight (Li et al., 2009; Chi et al., 2014). In recent years, as the demand for plant oils has sharply increased, improving the oil content and quality of oilseed crops has become a major goal globally.

Storage lipids, which mainly consist of triacylglycerol (TAG) synthesized from glycerol-3-phosphate (G3P) and fatty acids (FAs), generally accumulate during the maturation of seeds and are the major energy source for younger seedlings. In recent decades, many genes encoding key FA biosynthesis- or lipid reservoir-related enzymes or enzyme subunits from different organisms have been transformed into plants and increased the levels of lipids in transgenic seeds to varying degrees (Shorrosh et al., 1995; Zou et al., 1997; Jako et al., 2001). However, in some cases the oil content was reduced, for example, by overexpressing the gene encoding spinach 3-ketoacyl-ACP synthase III (KAS III) in transgenic rapeseed seeds (Dehesh et al., 2001). Nevertheless, FA synthesis and accumulation involve a highly coordinated regulatory network, not only associated with FA synthesis and lipid accumulation genes, but also some glycolysis related genes during carbon metabolism (Broun et al., 1999; Thelen and Ohlrogge, 2002; Jaworski and Cahoon, 2003; Cahoon et al., 2007; Weselake et al., 2009). Thus, manipulating only one or a few genes in the pathway of FA synthesis and metabolism sometimes resulted in discordant changes of FA composition and lipid contents.

Some recent studies found that several genes encoding embryo-specific transcription factors associated with embryogenesis and development, including *LEAFY COTYLEDON1* (*AtLEC1*), *LEC1-LIKE* (*AtL1L*), *LEC2*, and *WRINKLED1* (*WRI1*), could regulate the coexpression of certain genes encoding key enzymes involved in FA biosynthesis and TAG accumulation in seeds (Focks and Benning, 1998; Lotan et al., 1998; Stone et al., 2001; Kwong et al., 2003; Cernac and Benning, 2004; Mu et al., 2008; Peng and Weselake, 2011). The highly conserved CCAAT motifs for LEC1 (or L1L) binding are significantly enriched in promoters of all FA biosynthesis-related genes (Peng and Weselake, 2011). Overexpression of these genes increases the FA contents in the transgenic plants, accompanied by the enhanced expression of key FA synthetic genes and glycolytic genes (Cernac and Benning, 2004; Baud et al., 2007; Mu et al., 2008; Andrianov et al., 2010; Tan et al., 2011; Manan et al., 2017). However, overexpression of these genes via their constitutive expression or expression at a high level in seeds always resulted in deleterious traits or undesirable pleiotropic effects. For example, ectopic expression of the *AtLEC1* gene in the leaves of 35S:*LEC1* transgenic *Arabidopsis* induced the formation of an embryo-like structure and caused developmental abnormalities (Lotan et al., 1998). Moreover, the seed-specific expression of *ZmLEC1* under the control of the *EARLY EMBRYO PROTEIN* (*EAP1*) promoter significantly increased the embryo oil content of the resulting transgenic maize, but severely affected seed germination and leaf development of the transgenic plants. However, overexpression of *ZmWRI1*, a WRI1-like gene in maize (*Zea mays*) seeds, increased the oil content without producing detrimental agronomic traits (Shen et al., 2010). Furthermore, a recent study showed that seed-specific expression of *BnLEC1* and *BnL1L*, driven by two modified *Napin* A (*Nap*A) promoters that only have 18 and 4% of the full-length promoter activity, respectively, significantly enhanced oil accumulation in transgenic rapeseed seeds without producing any detectable abnormalities (Tan et al., 2011).

In peanut breeding, key goals have long been to increase the oil content and improve the FA composition in peanut seeds. In this paper, we describe that, under the control of full-length or 230-bp *Nap*A promoter, transgenic peanuts exhibiting seed-specific expression of *AtLEC1* grew and developed normally without any detrimental effects on major agronomic traits. In the developing seeds of the transgenic peanuts, the mRNA levels of a series of genes associated with FA biosynthesis and lipid accumulation were upregulated, the oil content and seed weight increased significantly, and the levels of major FAs changed markedly in all lines. These results provide an efficient approach for the genetic improvement of peanut seed oil production.

## Materials and Methods

### Plant Materials and Growth Conditions

The peanut cultivar “Fenghua No. 1” (FH1), as the subject of transformation, was kept and reproduced by our group. The FH1 plants and all corresponding transgenic plants were grown in our experimental field for transgenic research. Under the field conditions, seeds were usually sown in early May and harvested in late September. When grown on culture medium, all seeds were completely immersed in water for 30 min and then surface-sterilized with 75% ethanol for 1 min, followed by 0.1% mercury chloride for 15 min, and then rinsed four times with sterile water. The sterilized seeds were sown on 1/2 MS_0_ medium with 0.6% agar (Murashige and Skoog, 1962) and then cultured at 25°C under a 16 h/8 h light/dark cycle.

### Construction of Plant Expression Vectors

**FIGURE 1:**
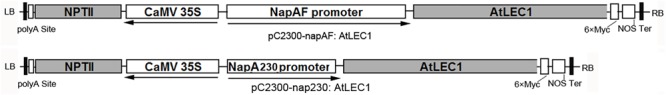
Structures of plant expression vectors harboring the foreign *AtLEC1* gene driven by Napin A full-length or 230-bp promoter.

The plasmids pC2300-*Nap*AF:*AtLEC1*(pF:*AtLEC1*) and pC2300-*Nap*A230:*AtLEC1*(p230:*AtLEC1*) (**Figure 1**) were kindly provided by Professor Jianru Zuo of the Institute of Genetics and Developmental Biology, Chinese Academy of Sciences. They are based on the pCAMBIA2300 framework with a *NEOMYCIN PHOSPHOTRANSFERASE II* (*NPTII*) gene for the selection of transformed plants, as well as the *AtLEC1* gene driven by a 1101-bp (full-length) or 230-bp promoter of *Nap*A from *Brassica napus* (*B. napus*), respectively. To facilitate the identification of foreign gene expression, a polypeptide tag of six × Myc was added at the C-terminus of AtLEC1.

### Generation of Transgenic Lines

The two plasmids mentioned above were transformed into *Agrobacterium tumefaciens* strain LBA4404, which was then used for peanut transformation. Using the epicotyl fragments of FH1 seeds germinated for 4 days as explants, transformation of peanut plants was carried out by an *Agrobacterium*-mediated method. A single colony of *Agrobacterium* harboring the target plasmid was propagated in 100 mL of liquid YEP medium containing 50 mg/L kanamycin at 28°C on a shaker for 24–36 h and reached the logarithmic growth phase (*A*_600_ at 0.8–1.0), and the bacterial cells were resuspended in an equal volume of liquid medium for shoot induction [MIS; MS medium plus 8 mg/L 6-benzylaminopurine (6-BAP), 0.7 mg/Lα-naphthylacetic acid (NAA), 30 g/L sucrose, pH 5.8]. The fresh explants were immersed in the suspension for 20 min, blotted dry on sterile filter paper to remove excess bacteria, and then transferred onto MIS solid medium and cultured in the dark at 25 ± 1°C for 48 h. The infected epicotyl explants were washed with MIS liquid medium containing 500 mg/L carbenicillin and cultivated on the selection MIS medium containing 50 mg/L kanamycin and 500 mg/L carbenicillin. After culture for 20–30 days, the induced adventitious buds were excised from the primary explants and subcultured on MSE medium for stem elongation (MS_0_ + 2 mg/L 6-BAP + 1.0 mg/L gibberellin GA_3_, 30 g/L sucrose, pH 5.8) containing the same screener and antimicrobial. When the kanamycin-resistant shoots reached approximately 5–6 cm in length, they were separated and grafted on the vigorous seedlings of wild-type peanut in a greenhouse.

The transgenic plants of the T_0_ generation were detected by polymerase chain reaction (PCR) and the sequencing of PCR products. Then, the positive transgenic plants of the T_1_–T_3_ generations were confirmed by PCR and at least 20 plants per line were detected. Using chi-squared test, the ratios of PCR-positive numbers to -negative numbers in every detected transgenic T_1_ line were assessed, the lines in accordance with Mendel’s first law were selected for generation, and the transgenic T_2_ and T_3_ progeny with positive PCR products were identified as homozygous lines to be used for further analysis. The PCR reaction conditions were as follows. The reaction mixture was predenatured for 5 min at 94°C, and then amplified for 35 cycles by denaturing for 25 s at 94°C, annealing for 30 s at 56°C, and extending for 45 s at 72°C, followed by final extension for another 5 min at 72°C. The forward and reverse primers for PCR were designed to match sequences in the promoter region and the ORF region of the target gene, respectively, with the following sequences: 5′-GTGATCGCCATGCAAATCTCC-3′ and 5′-CCAAGCTTGCTCATAGCCCA-3′.

### Protein Extraction and Western Blotting

Total soluble protein was extracted from the seeds at 30–40 days after pegging (DAP) of control FH1 and the transgenic T_3_ generation lines using a plant total protein extraction kit (Sangon Biotech Co., Ltd., Shanghai, China). Total protein extracts of over 40–50 μg for each sample were boiled for 5 min in 6× loading buffer (Transgen Biotech, Beijing, China), separated by 10% sodium dodecyl sulfate – polyacrylamide gel electrophoresis (SDS-PAGE), and then transferred to a 0.45-μm polyvinylidene difluoride (Roche Applied Science, Mannheim, Germany) membrane by the wet electrotransfer method (Tovey and Baldo, 1987). After transfer, blotting efficiency was checked by reversibly staining the transferred proteins with Ponceau S solution. Using mouse monoclonal antibody Myc-Tag (9B11; Cell Signaling Technology, Inc.) as the primary antibody, western blotting was carried out in accordance with the instructions of the Lumi-Light^PLUS^ Western Blotting Kit (Roche Applied Science).

### Analysis of Gene Expression by qRT-PCR

Total RNA was isolated from the immature seeds at about 15, 30, and 60 DAP for the control and transgenic plants by an improved version of the cetyltrimethylammonium bromide method (Chen et al., 2011). The first-strand cDNA synthesis was undertaken using oligo(dT) as a primer, in accordance with the instructions of the PrimeScript II 1st strand cDNA Synthesis Kit (Takara Biotechnology, Dalian, China). For real-time quantitative reverse transcription PCR (qRT-PCR) analysis, a diluted form of the RT reaction was used as a template and the amplification system was established with reference to the instructions of SYBR Premix Ex Taq (Tli RNaseH Plus; Takara Biotechnology, Dalian, China). The amplification was performed by predenaturation for 5 min at 94°C, and then 40 cycles of 15 s at 94°C and 30 s at 60°C. The relative expression levels of the target genes were analyzed with *AhACTIN7* as an internal control and calculated using the formulas *F* = 2^-ΔΔCt^ and ΔΔCt = (Ct mean value of the target gene in the sample - Ct mean value of the housekeeping gene in the sample) - (Ct mean value of the target gene in the control - Ct mean value of the housekeeping gene in the control). The detected genes and all corresponding primers are listed in Supplementary Table S1.

### Analysis of Seed Lipid Content and Fatty Acid Composition

The seed oil content was analyzed by the near-infrared reflectance (NIR) spectral method and the Soxhlet extraction method. NIR analysis of the oil content was carried out using a DA7250 NIR Analyzer (Perten Instruments, Hägersten, Sweden). The intact seeds from a single plant were scanned and at least six plants of each transgenic line and of the control were investigated.

For the Soxhlet extraction, over 2 g of seeds were oven-dried for 2 h at 105°C and then cooled to room temperature. The dried seeds were ground into a fine powder and then transferred into a preweighed bag made of Whatman 3 M filter papers (weight of filters and absorbent cotton recorded as M1), sealed, and weighed (M2). The bagged sample was transferred into a Soxhlet tube and extracted with about 100 mL of petroleum ether (boiling point 30–60°C) using Soxhlet Extractor R306 (Behr, Germany) for over 25 cycles. After the extraction, the bagged sample was dried in a preweighed round-bottomed flask (M3) at 80°C to evaporate the remaining petroleum ether, cooled, and weighed (M4). The oil content was calculated using the following formula: $M_{r}=\frac{M_{4}-M_{3}}{M_{2}-M_{1}}\times 100\%$ (Shivani et al., 2011). For each transgenic and control plant, the mean values of triplicate samples were calculated.

Analysis of FA content and composition was performed by gas chromatography (GC) using an HP-6890 (Agilent Technologies, Santa Clara, CA, United States) equipped with a capillary bonded fused-silica column, DB-23 (60 m × 250 μm × 0.25 μm; J&W Scientific, Rancho Cordova, CA, United States). The sample was treated as previously described (Tang et al., 2012). Each FA methyl ester (FAME) sample was analyzed three times. The analytical conditions were as follows: the initial column temperature was 180°C, which was subsequently increased by a set program. The amount of added sample was 1 μL. The injector and detector temperatures were 260 and 270°C, respectively, the N_2_ gas flow rate was 2.0 mL min^-1^, and the split ratio was 30:1. FA content was calculated as absolute content (mg/g) using the GC area, based on the quantification of known amounts of internal standards. The quantities of the FAMEs in each sample were calculated using the following formula: W_i_ = A_i_M_s_/A_s_M, where *M*_s_ is the weight of the internal standard mixed into a sample, *A*_i_ is the area of the individual FAME, *A*_s_ is the area of the corresponding FAME in the internal standard, and *M* is the sample weight.

### Investigation of Agronomic Traits

The transgenic lines and their untransformed control were planted in adjacent fields. The seedling rate, plant height, branch number per plant, and flowering time were investigated during the vegetative development stage. Other traits including the number of pods per plant, the average pod and seed weights, pod shape, seed shape, and seed coat color were evaluated after harvest.

### Statistical Analysis

All experiments were performed with three replicates. Student’s *t*-test in SPSS (Statistical Product and Service Solutions) was applied to identify the differences between the transgenic lines and their control in oil content, seed and pod weight, and FA content, and *p* < 0.05 and *p* < 0.01 were considered to indicate significant differences. One-way analysis of variance (ANOVA) for gene expression levels among different transgenic peanut lines and their control at different developmental stages was performed by SPSS software, and the significance of differences (*p* < 0.05) was assessed by Duncan’s new multiple range test with values with different letters being significantly different.

## Results

### Generation and Characterization of Transgenic Peanut Plants Carrying *AtLEC1* Gene

**FIGURE 2:**
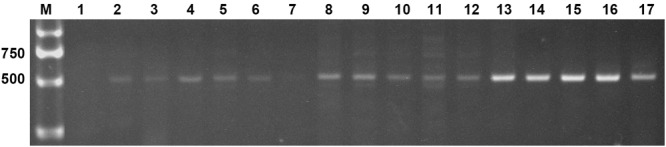
Polymerase chain reaction (PCR) amplification of the foreign fragment in transgenic peanut plants and untransformed control. M, DL2000 DNA markers (Takara); 1, untransformed control; 2–17, PCR products from transgenic plants.

Two expression constructs, p230:*AtLEC1* and pF:*AtLEC1*, were transformed into the peanut cultivar FH1 by *A. tumefaciens*-mediated transformation. Multiple independent transformed plants with kanamycin resistance were obtained. Specific bands of ∼500 bp including 63-bp *Napin* A promoter fragment and of ∼450 bp including the *AtLEC1* segment were observed upon PCR analysis of the transgenic plants, whereas the untransformed control showed no amplification (**Figure 2**). The PCR products were confirmed by sequencing. A total of 15 transgenic plants of the T_0_ generation were obtained.

Out of 15 T_0_ plants, only 12 yielded seeds, which were used to produce the next generation. The positive transgenic plants of the T_1_–T_3_ generations were confirmed by PCR. Nine T_1_ plants carrying the p230:*AtLEC1* construct and 12 T_1_ plants harboring the pFL:*AtLEC1* construct were yielded and used to create T_2_ progeny. According to the chi-square test, the T_2_ lines were all in accordance with the segregation ratio of Mendel’s first law. Thus, T_2_ transgenic lines with single-copy insertion were obtained. These transgenic lines were derived from six transformation events (three events 030801, 031001, and 031607 with the 230-bp promoter, and another three events 040201, 040305, and 040603 with the full-length promoter) and planted in a greenhouse. The plants that were developing well and contained foreign genes were selected and then planted in a greenhouse to generate T_3_ homozygous transgenic lines. Four homozygous transgenic lines (030801-3-5, 031001-2-3, 040201-11-8, and 040603-3-9) from two transformed constructs were used for further analysis.

### Analysis of Foreign Gene Expression by qRT-PCR and Western Blotting

**FIGURE 3:**
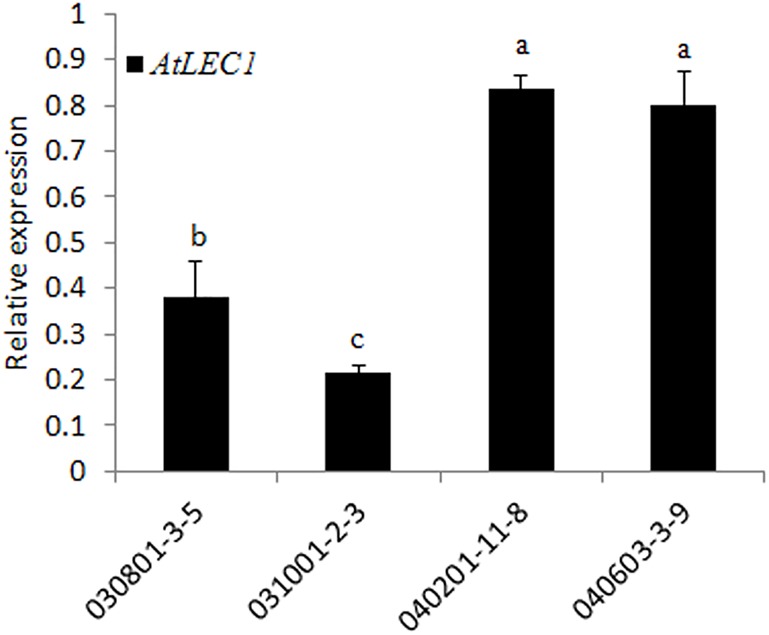
Transcript level analysis of the foreign *AtLEC1* gene in different transgenic peanut lines by qRT-PCR. One-way analysis of variance (ANOVA) for gene expression levels between different transgenic peanut lines was performed by SPSS software, and the significance of differences (*p* < 0.05) was assessed by Duncan’s new multiple range test with values with different letters being significantly different.

To check the transcription level of *AtLEC1* in different transgenic lines, qRT-PCR analysis was performed by the SYBR green method using the specific primers for *AtLEC1*. The results showed that the expression levels of *AtLEC1* in seeds differed among the different transgenic lines, and the accumulation of mRNA in the lines with the FL *Nap*A promoter was higher than for those with the 230-bp promoter (**Figure 3**), suggesting that the FL promoter had higher transcriptional activity than the truncated one.

**FIGURE 4:**
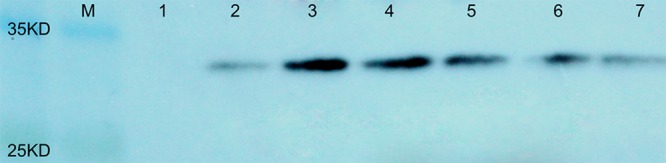
Western blotting of the AtLEC1–MYC fusion protein in seeds of different transgenic peanut lines. M, prestained protein ladder (180, 135, 100, 75, 63, 48, 35, 25, 17, and 11 kDa; Thermo Scientific); 1, untransformed control; 2–7, different transgenic peanut lines (040305-6-5, 040201-11-8, 040603-3-9, 030801-3-5, 031001-2-3, and 031607-2-4, respectively).

To evaluate the protein content in the seeds of transgenic plants containing *AtLEC1*, total soluble proteins were extracted from about 0.5 mg of immature seeds at 30–40 DAP, and 40–50 μg of seed protein of each transgenic plant was separated by SDS-PAGE and used for western blotting. It was predicted that the molecular weight of the fusion protein of AtLEC1 and Myc tag is about 32 kDa. Using the Myc-Tag monoclonal antibody from mouse as the primary antibody, in western blotting, distinctive hybridization signals were detectable in the seeds of different transformed plants (**Figure 4**).

### Analysis of Seed Oil Content and Weight

Using the NIR method, the seed oil content and major FA composition of 21 T_2_ transgenic lines were analyzed. Compared with that of the non-transgenic FH1, 15 out of 21 analyzed transgenic lines had a remarkably enhanced seed oil level (data not shown). To measure the oil content precisely, four T_3_ transgenic lines (with each line representing a transformation event) with significant increases of oil content were selected and analyzed by the standard Soxhlet extraction method. Under the experimental conditions, the oil contents of the transgenic seeds ranged from 55.07 to 61.09%, while that of non-transgenic control seeds was only 52.71%, and they increased by 4.42–15.89% in comparison to the oil content of FH1 (**Table 1**).

**Table 1:** Oil content in T_3_ transgenic peanut seeds and FH1.

Genotype	Line	Oil content (% dry weight)	Percentage of increase
WT	FH1	52.71 ± 0.83	–
p230:*AtLEC1*	030801-3-5	58.33 ± 1.00^∗∗^	10.64
	031001-2-3	55.07 ± 0.53^∗^	4.42
pF:*AtLEC1*	040201-11-8	61.09 ± 0.79^∗∗^	15.89
	040603-3-9	58.27 ± 0.93^∗∗^	10.53

*Mature seeds were used for the analysis of the oil content by the Soxhlet method. Asterisks indicated statistically significant differences compared with the control (Student’s t-test: ^∗^p < 0.05, ^∗∗^p < 0.01).*

Similar to the level in the control FH1, the transgenic lines produced 20–35 cocoon-shaped pods per plant, and over 90% of them have two seeds per pod. There were not any visible changes in pod and seed size in transgenic peanuts compared to the untransformed control. A total of 20 full pods and 20 seeds were taken from each transgenic line and weighed to calculate the average weight per pod and per seed (**Table 2**). Compared with those in the control, the average weights of pods and seeds of the T_3_ transgenic lines increased by 2.9–14.9% and 11.1–22.2%, respectively.

**Table 2:** The Average weight of pods and seeds in T_3_ transgenic peanut lines.

Genotype	Line	Average pod weight (g)	% Increase over control	Average seed weight (g)	% Increase over control
WT	FH1	2.42 ± 0.08	–	0.81 ± 0.08	–
p230:*AtLEC1*	030801-3-5	2.78 ± 0.03^∗∗^	14.9	0.93 ± 0.03^∗∗^	14.8
	031001-2-3	2.49 ± 0.02^∗^	2.9	0.90 ± 0.02^∗∗^	11.1
pFL:*AtLEC1*	040201-11-8	2.58 ± 0.05^∗^	6.6	0.92 ± 0.05^∗∗^	13.6
	040603-3-9	2.73 ± 0.09^∗∗^	12.8	0.99 ± 0.09^∗∗^	22.2

*At least 20 full pods and 20 seeds from each transgenic line were weighed. Asterisks indicated statistically significant differences compared with the control (Student’s t-test: ^∗^P < 0.05, ^∗∗^P < 0.01).*

### Analysis of FA Contents and Composition in Transgenic Seeds

The FA contents and composition of control FH1 and transgenic seeds were analyzed by GC. The results showed that, compared with their levels in FH1 seeds, FA C18:0 and C18:1n9 were significantly increased in transgenic seeds, whereas the C16:0 and C18:2n6 levels were significantly decreased. The most markedly changed FAs were unsaturated C18:1 and C18:2 in the transgenic lines 040201-11-8 and 040603-3-9 (**Table 3**). The greater increase of C18:1 and decrease of C18:2 resulted in elevation of the ratio of oleic acid (C18:1) to linoleic acid (C18:2, O/L) in these lines. Except in line 031001-2-3, the levels of the major long-chain FAs (C20 and longer chains) in transgenic lines were decreased to varying degrees (**Table 3**). Among those long-chain FAs, erucic acid (C22:1n9) is a key factor adversely affecting the quality of edible oil. Peanut cultivars including FH1 generally contain very low C22:1n9, and all transgenic lines similar to FH1 also have a low C22:1n9 level.

**Table 3:** Comparison of FA composition and relative FA contents in mature seeds between FH1 and transgenic peanut lines (%).

Fatty acid	Line				
	030801-3-5	031001-2-3	040201-11-8	040603-3-9	FH1
C16:0	12.44 ± 0.00^∗^	12.31 ± 0.02^∗^	10.12 ± 0.03^∗∗^	10.26 ± 0.00^∗∗^	13.04 ± 0.06
C16:1	0.05 ± 0.00	0.07 ± 0.00	0.06 ± 0.00	0.05 ± 0.00	0.07 ± 0.00
C18:0	3.44 ± 0.01^∗∗^	3.51 ± 0.00^∗∗^	4.05 ± 0.01^∗∗^	4.00 ± 0.04^∗∗^	2.93 ± 0.01
C18:1n9	40.48 ± 0.02^∗^	38.55 ± 0.05^∗^	52.06 ± 0.01^∗∗^	49.65 ± 0.00^∗∗^	37.3 ± 0.03
C18:2n6	37.98 ± 0.03^∗^	38.44 ± 0.02^∗^	28.89 ± 0.02^∗∗^	30.91 ± 0.10^∗∗^	40.62 ± 0.12
C18:3	0.04 ± 0.00	0.05 ± 0.00	0.04 ± 0.00	0.06 ± 0.00	0.06 ± 0.00
C20:0	1.46 ± 0.00	1.49 ± 0.00	1.40 ± 0.00	1.43 ± 0.01	1.40 ± 0.01
C20:1	0.68 ± 0.00^∗^	0.86 ± 0.00^∗∗^	0.64 ± 0.01^∗∗^	0.72 ± 0.01	0.76 ± 0.00
C22:1n9	0.03 ± 0.00	0.04 ± 0.00	0.03 ± 0.00	0.03 ± 0.00	0.04 ± 0.00

*Mature seeds were used for the analysis of the FA contents and composition by GC. Data are presented as the mean values of three independent detections. Asterisks indicated statistically significant differences compared with the control (Student’s t-test: ^∗^p < 0.05, ^∗∗^p < 0.01).*

### Transcriptional Analysis of Several Genes Associated With FA Synthesis and Lipid Accumulation

**FIGURE 5:**
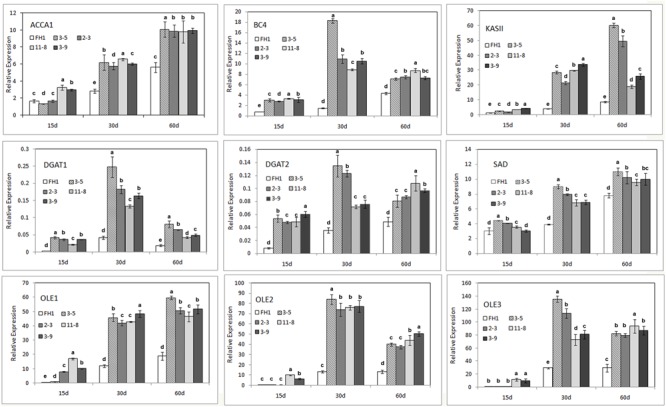
Expression analysis of nine endogenous genes involved in FA synthesis and lipid accumulation in the developmental seeds of different transgenic peanut lines, as measured by qRT-PCR. One-way analysis of variance (ANOVA) for gene expression levels among different transgenic peanut lines and their control at different developmental stages was performed by SPSS software, and the significance of differences (*p* < 0.05) was assessed by Duncan’s new multiple range test with values with different letters being significantly different. FH1, untransformed control. Relative transcript levels were quantified by comparison to that of peanut actin7 as a reference gene.

The majority of FA synthesis and lipid deposition in developing peanut seeds generally occurs around 10–70 DAP (data not shown). The full-length and 230-bp promoters of the *Nap*A gene from *B*. *napus* were associated with increased expression from the heart stage to the cotyledon stage (10–30 days after pollination in rape) of seeds (Stålberg et al., 1993). Thus, we obtained immature seeds at three stages (15, 30, and 60 DAP) as the materials for analysis and subjected them to qRT-PCR. The genes analyzed include the key genes in the FA *de novo* synthetic pathway [the genes encoding homomeric ACCase A (*AhACC II*), the BCCP subunit and BC subunit of heteromeric ACCase (*AhBCCP1* and *AhBC4*), ketoacyl-ACP synthetase (*AhKAS II*), and the gene encoding stearoyl-ACP desaturase (*AhSAD*)], as well as genes involved in lipid accumulation, such as those encoding diacylglycerol acyltransferase and oleosin (*AhDGAT1, AhDGAT2, AhOle1, AhOle2*, and *AhOle3*). Except for *AhBCCP1*, the expression of all genes mentioned above was enhanced to varying degrees in the seeds of transgenic lines at all three seed developmental stages (**Figure 5**). At the 15 DAP stage, compared with those in the untransformed control, the levels of the transcripts of these genes in seeds increased; their transcription levels were lower than those at other development stages, for both transgenic lines and controls. In comparison with the control, the mRNA abundance in the seeds of transgenic lines at 30 and 60 DAP was remarkably increased, and for some genes particularly at 30 DAP (**Figure 5**).

**FIGURE 6:**
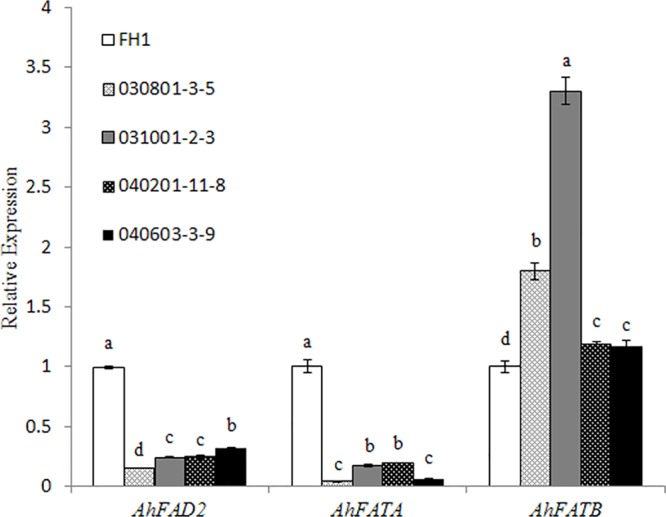
Expression analysis of *AhFAD2, AhFATA*, and *AhFATB* in the seeds of different transgenic lines at 30 DAP by qRT-PCR. One-way analysis of variance (ANOVA) for gene expression levels among different transgenic peanut lines and their control was performed by SPSS software, and the significance of differences (*p* < 0.05) was assessed by Duncan’s new multiple range test with values with different letters being significantly different FH1, untransformed control. Relative transcript levels were quantified by comparison to that of peanut actin7 as a reference gene.

Owing to significant variation in the levels of C18:1, C18:2, and C16:0, we checked the transcript changes of Δ^12^ FA desaturase (FAD2), and acyl-ACP thioesterases FATA and FATB related to the accumulation of the above FAs in the seeds of transgenic plants and the control at 30 DAP. It was found that the mRNA accumulation of FAD2 and FATA was decreased in the transgenic seeds with the two promoters of different lengths, while the transcript level of FATB increased in comparison with that in the control (**Figure 6**).

## Discussion

### *AtLEC1* Overexpression in Peanut Seeds Improved the Oil Content and Weight of Seeds, While Not Altering the Major Agronomic Traits

**FIGURE 7:**
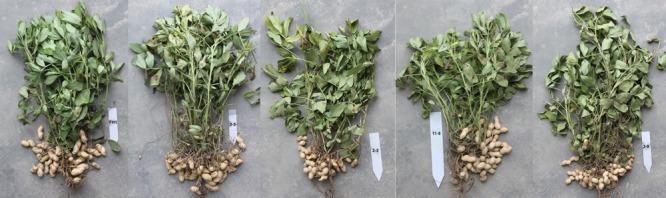
Phenotype of transgenic peanut and its untransformed control at the harvest stage.

Improving the oil content of peanut seeds has consistently been a target of peanut breeding programs. Against this background, we transferred a key regulator of FA biosynthesis, the *AtLEC1* gene, into peanut seeds and obtained several transgenic lines with higher oil content and heavier seeds than the untransformed control. These transgenic plants harboring the full-length or truncated 230-bp seed-specific *Nap*A promoter did not show any visible developmental abnormalities regarding major agronomic traits. Previous studies indicated that the overexpression of some seed development-related genes, such as *LEC1, LEC1-like*, and *WRI1*, via either constitutive expression or expression at a higher level in dicots and monocots could increase the oil content, but also led to a series of disorders of agronomic traits (Mu et al., 2008; Shen et al., 2010; Tan et al., 2011). For example, the overexpression of *AtLEC1* by an estradiol-inducible promoter (pER8) caused an elevated FA level in transgenic *Arabidopsis* seedlings. A similar phenotype was observed in transgenic *Arabidopsis* overexpressing LEC1-like genes of *B. napus*. However, when germinated and grown in the presence of estradiol, transgenic seedlings showed markedly reduced growth (Mu et al., 2008). Moreover, the overexpression of *ZmLEC1* under two promoters that preferentially function at the embryonic stage, a strong *OLEOSIN* (*OLE*) promoter and a weaker *EARLY EMBRYO PROTEIN* (*EAP1*) promoter, similarly increased the seed oil accumulation and embryo size, but caused reductions in seed germination and leaf growth (Shen et al., 2010). The undesirable phenotypes in transgenic lines produced by the strong *OLE* promoter were more serious than those by the weaker *EAP1* promoter (Shen et al., 2010). In our study, two seed-specific promoters, namely, the full-length 1101-bp *Nap*A and the truncated 230-bp *Nap*A, were used. The profiles of their expression reveal the characteristics of developmental dependence and spatial specificity, similar to those in embryos of transgenic tobacco, with expression being barely detectable 5–10 days after pollination (globule to heart stages), but with significant enhancement at 10–15 days (heart to torpedo stages), and reaching the highest expression level at 30 days (maturation stage) (Stålberg et al., 1993; Ellerström et al., 1996; Tan et al., 2011). The 1101-bp promoter was found to direct β-GLUCURONIDASE (GUS) synthesis in the transgenic tobacco embryos at a level approximately 10-fold higher than the 35S promoter (Stålberg et al., 1993), and the 230-bp promoter lost more than 82% of the 1101-bp promoter’s activity (Tan et al., 2011). We found that the expression patterns of all genes in the transgenic peanut seeds were basically consistent with the performance of the two promoters. That is, the transcript levels of most genes in transgenic 30- and 60-DAP seeds harboring the 1101-bp *Nap*A promoter were higher than those with the 230-bp promoter. However, irrespective of whether the full-length *Nap*A or 230-bp promoter was used, the transgenic lines exhibited normal traits similar to those of the control FH1 (**Figure 7**). Regarding these similarities to the agronomic characteristics of FH1, the height of these lines was also about 43 cm, and they had inverted oval-shaped darker-green leaves, 9–10 branches of stems, and produced 20–35 cocoon-shaped pods in each plant. In addition, there were not any visible changes in pod and seed size in transgenic peanuts compared to FH1. In all transgenic peanut seeds, oil contents increased significantly, whereas protein contents remained unchanged. Therefore, it was suggested that the increase of seed weights was attributable to an enhancement of TAG accumulation. Thus, our results conflict with those of transgenic *Arabidopsis* reported by Tan et al., who found that transgenic plants overexpressing *BnLEC1* controlled by the full-length *Nap*A promoter exhibited severe abnormality after germination, and some plants died before flowering or did not produce seeds. Our results led to the conclusion that the FA biosynthesis-related genes in our *AtLEC1* transgenic peanut plants with the 1101-bp promoter were reasonably upregulated.

### The Distinct Change of Main FA Contents in the Transgenic Seeds Is Due to Some Genes Involved in FA Modification and TAG Accumulation Expressed Synergistically

In higher plants, the biosynthesis of FA and TAG has been well characterized by biochemical and molecular biology approaches. FA is synthesized *de novo* mainly from acetyl-CoA in plastids by a repeated series of reactions, including the carboxylation catalyzed by ACCase, and the continuous condensation, reduction, and dehydration reactions accompanied by the two-carbon chain length extension in each cycle, resulting in major acyl-ACPs such as 16:0-ACP, 18:0-ACP, and 18:1-ACP. Then, by the thioesterases FATA and FATB, the majority of acyl moieties are hydrolyzed from ACP and released into the acyl-CoA pool in the cytoplasm. Subsequently, the desaturation and elongation of FA acyl chains are performed by different FA desaturases (FADs) and elongases (FAEs) in plastid or in endoplasmic reticulum. Finally, acyl chains are added at the sn1-3 sites of the G3P backbone by a series of acyltransferases and form the TAG (Ohlrogge and Browse, 1995; Voelker and Kinney, 2001; Sasaki and Nagano, 2004). In recent years, several studies have indicated that TAG biosynthesis pathways include the acyl-CoA-dependent Kennedy pathway, the acyl-CoA-independent pathway, and a novel monoacylglycerol pathway (Yu et al., 2015). Thus, the FA composition and content in TAG not only rely on the components of the acyl-CoA pool, but are also involved in the network of acyl fluxes (Chen et al., 2015; Yu et al., 2015; Bates, 2016; Woodfield et al., 2017).

Substantial diversity in oil storage is observed in terms of the level of accumulation, the rate of oil synthesis, and the TAG structure in the reservoir tissues of different plant species, which is displayed in the assembly pathways of TAG and the different functions of corresponding enzymes (Peng and Weselake, 2011; Troncoso-Ponce et al., 2011; Yu et al., 2015). In *Arabidopsis* and *B. napus*, DGAT1 is the predominant enzyme in TAG synthesis, which catalyzes the accumulation of normal TAG with a high proportion of erucic acid (C22:1) in seeds, whereas in *Ricinus communis*, DGAT2 may be important for incorporation of the hydroxylated acyl into TAG (Zou et al., 1999; Troncoso-Ponce et al., 2011). Peanut seeds are enriched in oleic acid and linoleic acid, which account for 36–67% and 15–43% of the oil content of seeds, respectively. AhDGAT1 was considered to be the key enzyme for synthesizing TAG in seeds, which preferentially incorporates unsaturated C18 FAs into TAG (Chi et al., 2014; Zheng et al., 2017). In our transgenic peanuts overexpressing *AtLEC1*, compared with that in the control, *AhDGAT1* expression was significantly enhanced at three developmental stages of seeds, resulting in a remarkable increase of the oil contents of transgenic seeds, where the C18 unsaturated FAs were the major components. In fact, lipid accumulation in seeds presents a range of distributions among the various tissue types, and the lipid profiles also have tissue or spatial specificity. The embryonic axis was particularly enriched in palmitic acid, while the cotyledon mainly accumulated more oleic acid (Horn et al., 2012, 2013; Woodfield et al., 2017). In our transgenic lines, the FL or 230-bp *Napin* A promoter had higher activity in cotyledons prior to formation of the embryo axis (Stålberg et al., 1993; Ellerström et al., 1996). Thus, the C16 FAs in transgenic seeds accounted for a lower proportion of the total than in the control.

Woodfield et al. (2017) reported that the discrepant pattern of FA composition between the embryonic tissues and the cotyledons could be attributable to a spatial difference in the expression of *FATA* and *FATB*. In general, the thioesterase FatA has higher *in vitro* activity for 18:1-ACP and lower activities for 18:0-ACP and 16:0-ACP substrates, while FatB preferentially acts on saturated FAs containing 8–18 carbons (Salas and Ohlrogge, 2002; Wallis and Browse, 2002). In the *fata* mutant of *Arabidopsis*, a reduction of *FATA* was found to affect the ratio between C16 and C18 FAs, and caused a drop of palmitic acid incorporated into glycerolipids, but no apparent changes of C16 FA in seeds. In contrast, in *Arabidopsis fatb* mutant, the relative C16:0 FA content was significantly decreased in all tissues including seeds, and C18:0 content also decreased in seeds (Bonaventure et al., 2003, 2004). In peanut, there were some differences in the profiles of substrates hydrolyzed by FATB. Ectopic expression of *AhFATB1* in *Escherichia coli* effectively enhanced the levels of myristic acid (14:0), palmitoleic acid (16:1), and oleic acid (18:1), while the level of palmitic acid (16:0) decreased (Chen et al., 2012). In our results, the expression level of *AhSAD* markedly increased in transgenic seeds, which catalyzed more 18:0-ACP molecules to form 18:1-ACP. In addition, the increase of *AhFATB1* expression could release more 18:1-ACP molecules into the acyl-CoA pool and further participate in the synthesis of TAG. In addition, suppression of the *AhFAD2* transcriptional level caused an increase of 18:1 by a reduction of the synthetic flux of 18:2. Therefore, the changes in the expression of these genes resulted in a significant increase of oleic acid (18:1) content in the seeds of all transgenic lines, and a corresponding reduction of the linoleic acid (18:2) content.

## Conclusion

The overexpression of *AtLEC1* in a seed-specific pattern in peanut increased the oil content and seed weight, and altered the FA composition of seeds by coordinately regulating the expression of several FA synthesis- and TAG accumulation-related genes. Moreover, there were no major changes in the main agronomic traits in all analyzed transgenic lines. This study provides a reasonable approach for the genetic improvement of peanut seed oil production.

## Author Contributions

LS and SW: conceived and designed the experiments. GT, PX, WM, and FW: performed the experiments. GT, PX, WM, ZL and LS: analyzed the data. GT, ZL, and LS: wrote the paper. All authors revised the draft and approved the final manuscript.

## Conflict of Interest Statement

The authors declare that the research was conducted in the absence of any commercial or financial relationships that could be construed as a potential conflict of interest.
