# Supplementary material for: Seed-Specific Expression of AtLEC1 Increased Oil Content and Altered Fatty Acid Composition in Seeds of Peanut (Arachis hypogaea L.)
Source: Front Plant Sci. 2018 Mar 6;9:260. doi: 10.3389/fpls.2018.00260 (PMC5845668; doi:10.3389/fpls.2018.00260)
Supplement: Supplementary file 1 [file Table_1.docx]

Table S1. All corresponding primers used for qRT-PCR analysis

| Gene | Forward primer | Reverse primer | Size of PCR products（bp） |
| --- | --- | --- | --- |
| *AhBCCP1* | CTTCTTCCGCATCTTCTTCG | GTAGGAGCGACAGCATTTG | 221 |
| *AhaccA2* | GCGTTTGCAAGATCAGAACA | CCGACTTCAAAGACTCAGCC | 230 |
| *AhBC4* | AGACCAGGGCCAGGTAGAAT | GGAGCCCACACAATCAACTT | 137 |
| *AhKASII* | TCTATCAGCGGCCAGAAAGT | AACAATGTCCATGTCCCCAT | 105 |
| *AhSAD* | AATCGTTAGAGGGTTGGGCT | TTTTGCCCTTGCTCTCAGTT | 143 |
| *AhFAD2* | CGACCGCAACGAAGTGTT | CCCTCCCTGGTGGATTGT | 82 |
| *AhFATA* | TATTCCAGGCTTAGACTCAT | CTCAAGAACCCAACCAAT | 90 |
| *AhFATB* | GAAGAAGTCAGAGCGGAGAT | GACCCGTGCGAATGTAAT | 118 |
| *AhDGAT1* | CAACACGTCACCAATTTTGC | TATCGTCGGAGCTTGAATCC | 231 |
| *AhDGAT2a* | CAGGGTTTATGCCTCTTCCA | GCATGGCTATGCGGATAAAT | 249 |
| *AhOle1* | GCTCTTCGTGATCTTCAGCC | CCAGGTGGATGCTTACCTGT | 153 |
| *AhOle2* | CCGCTTTTCATCCTCTTCAG | CCGGATGTAATTCATGACCC | 119 |
| *AhOle3* | AGTCACCAGCCACCAAAAAC | GGGGTCATAGGAGGTTCCAT | 137 |
| *AtLEC1* | GGCAGAGAAACAATGGAACGT | TGTGCTGGTCCAAGTTCAATTCA | 85 |
| *AhACT7* | ATGTATGTAGCCATCCAAG | ACCAGAGTCCAGAACAATA | 75 |
